# Research on Positioning Accuracy of Mobile Robot in Indoor Environment Based on Improved RTABMAP Algorithm

**DOI:** 10.3390/s23239468

**Published:** 2023-11-28

**Authors:** Shijie Zhou, Zelun Li, Zhongliang Lv, Chuande Zhou, Pengcheng Wu, Changshuang Zhu, Wei Liu

**Affiliations:** Chongqing University of Science and Technology, Chongqing 401331, China; 2021203255@cqust.edu.cn (S.Z.); 2010024@cqust.edu.cn (Z.L.); 2007004@cqust.edu.cn (C.Z.); 2022203042@cqust.edu.cn (P.W.); 2022203050@cqust.edu.cn (C.Z.); 2022203101@cqust.edu.cn (W.L.)

**Keywords:** visual SLAM, RTABMAP, robot localization, multi-sensor fusion

## Abstract

Visual simultaneous localization and mapping is a widely used technology for mobile robots to carry out precise positioning in the environment of GNSS technology failure. However, as the robot moves around indoors, its position accuracy will gradually decrease over time due to common and unavoidable environmental factors. In this paper, we propose an improved method called RTABMAP-VIWO, which is based on RTABMAP. The basic idea is to use an Extended Kalman Filter (EKF) framework for fusion attitude estimates from the wheel odometry and IMU, and provide new prediction values. This helps to reduce the local cumulative error of RTABMAP and make it more accurate. We compare and evaluate three kinds of SLAM methods using both public datasets and real indoor scenes. In the dataset experiments, our proposed method reduces the Root-Mean-Square Error (RMSE) coefficient by 48.1% compared to the RTABMAP, and the coefficient is also reduced by at least 29.4% in the real environment experiments. The results demonstrate that the improved method is feasible. By incorporating the IMU into the RTABMAP method, the trajectory and posture errors of the mobile robot are significantly improved.

## 1. Introduction

In recent years, the demand for high-precision mapping and positioning has significantly increased due to the advancement and evolution of autopilot, AR and VR, UAV, and robotics. Mapping and positioning are crucial components in various innovative technologies, and their accuracy and robustness are of great importance. The field of simultaneous localization and mapping (SLAM), also known as Concurrent Mapping and Localization (CML), is a popular area of study for scholars both domestically and internationally. SLAM is used to process and construct maps in unknown environments and to determine the location of robots within these maps [1,2,3,4]. Currently, the construction of unknown environment maps and the acquisition of location information primarily rely on sensors such as cameras (mono, stereo, RGB-D), lidar (single, multi-beam), and sonar (mainly used for underwater mapping). Although lidar is widely used in real life, the advantages of low cost, rich semantic information, high-resolution images, and other advantages of camera solutions have always been popular areas of research.

In fact, it is not easy to construct an efficient and accurate map and obtain its own precise location in it [5]. It requires a variety of different information. Single sensors often struggle to meet the requirements for precise positioning due to limitations imposed by their working principles and environmental conditions [6]. To overcome these limitations, multi-sensor fusion positioning has become an area of great interest.

Mourikis et al. [7] proposed the VI-SLAM framework MSCKF in 2007. This method integrates IMU and camera data within the Extended Kalman Filter (EKF) [8] framework. The key contribution of this method is the development of a measurement model that can express the geometric constraints that arise when observing static features from multiple camera positions [9]. This model can adapt to more aggressive motion and texture loss over a certain period of time, and serves as the foundation for many current VI-SLAM systems. Bloesch et al. [10,11] proposed a direct monocular visual inertial odometry method (ROVIO) based on the Iterative Extended Kalman Filter (IEKF). The main idea of this method is to track multi-level image features using pixel intensity errors of image blocks and directly incorporate them into the EKF update step. This results in more accurate tracking performance. This method also employs a robocentric approach and a minimal representation of landmark parametrization to enhance consistency and computational efficiency. Leutenegger et al. [12] proposed a method to tightly integrate inertial measurement values into keyframe-based visual SLAM (OKVIS). The primary task is to closely integrate visual measurements and IMU readings by using a joint nonlinear cost function. This approach enables better utilization of information from both sensors and takes into account IMU errors during optimization. It maintains global consistency even when the system is stationary or moving slowly. To ensure real-time operation, they maintain a bounded-sized optimization window by partially marginalizing the old state. Qin et al. [13] proposed a method for VINS-Mono (monocular visual system) that utilizes a sliding-window-based optimization approach to combine monocular vision and IMU data. The primary objective of this method is to utilize previous information to constrain state estimation and enhance the accuracy of the results; also, this approach enables the attainment of more precise state estimation results while maintaining computational efficiency. Mur-Artal et al. [14,15] proposed ORB-SLAM2 in 2016. The front-end visual odometry of this method uses “Oriented FAST” key points and BRIEF descriptors to extract and match feature points, while the back-end uses nonlinear optimization with the BA method. The ORB feature points and bag-of-words method are utilized for feature extraction and matching, providing benefits such as illumination and scale invariance, as well as fast matching speed. Additionally, an incremental map construction method is implemented, allowing for real-time dynamic map updates and support for various camera types, including monocular, stereo, and RGBD cameras. This approach has become a classic work in the field of SLAM due to its numerous advantages. Stumberg [16] proposed a monocular visual inertial odometry method based on delayed marginalization and attitude beam adjustment, DM-VIO (Differential Monocular Visual-Inertial Odometry). The main work of this paper includes the proposal of a delayed marginalization strategy to optimize factor graphs in sliding window optimization. Conventional VIO methods usually use sliding window optimization to estimate status, but the frequent edging of the old state will cause the density of graphs to decrease [17]; therefore, DM-VIO suggests retaining some older keyframes without marginalization to maintain graph density. They also use reparameterization to minimize the impact of delayed marginalized frames on the most recent frame.

Labbé et al. [18] proposed RTABMAP, which is an SLAM algorithm based on probabilistic graph optimization. RTABMAP supports the simultaneous input of camera and lidar data and allows users to simultaneously output 3D dense point cloud maps and 2D grid occupancy maps. This method divides all nodes at runtime into three categories, STM (short-term memory), WM (working memory), and LTM (long-term memory). Its unique hierarchical memory management mechanism can coordinate the planning and modeling of input image frames. While controlling the amount of mapping calculations within the tolerance of the device, it can also achieve high real-time performance and loopback detection accuracy.

In indoor SLAM, cameras have an advantage over 3D lidar in terms of cost and semantic information. Meanwhile, wheel odometry is also one of the common sensors in mobile robots. Table 1 shows the comparison of the above-mentioned SLAM methods, from which it can be seen that RTABMAP not only supports the above three sensors, but also outputs 2D grid occupancy maps and 3D dense point cloud maps at the same time. This makes it possible to allow users to access different sensors and robots to develop various solutions, and also makes it one of the classic SLAM algorithms.

However, we found that RTABMAP’s own localization accuracy in the constructed maps is not very high in practical tests, especially in the environment with multiple interference factors. So, in this paper, we hope to improve the robustness and real-time performance of the RTABMAP algorithm by improving the algorithm to adapt to diverse scenes. At the same time, it provides a new reference for research and application in the field of SLAM. This paper aims to optimize the RTABMAP method and integrate data from various sensors. By combining the strengths of different sensors, their weaknesses can be compensated for, resulting in more comprehensive, accurate, and reliable positioning results. This article makes the following main contributions:

1. An improved algorithm for RTABMAP is presented in this paper. The main idea is to use an EKF to fuse the IMU with the wheel odometry and provide a new state estimation model to achieve more accurate location estimation.
2. We have validated the improved method through experiments conducted in public datasets (indoors) as well as real environments, which include enclosed rooms and open corridors.

## 2. Related Work

### 2.1. IMU State Estimation Model

The inertial measurement unit (IMU) mainly consists of sensors such as accelerometers, gyroscopes, magnetometers, and others. It is generally categorized as 9-DOF and 6-DOF (excluding the magnetometer). In addition to violent vibrations and shocks, IMUs are hardly disturbed by other forms of external signals and extreme weather and strong light. Due to the redundancy between the measured values of angular velocity and linear acceleration, the “relative position change in a short period of time” of the IMU output has a very high degree of confidence. The IMU is an independent system that can estimate the relative position without relying on any external device to assist the derivation. In contrast, lidar, camera, etc., are dependent on external sensing and have some instability.

**Figure 1:**
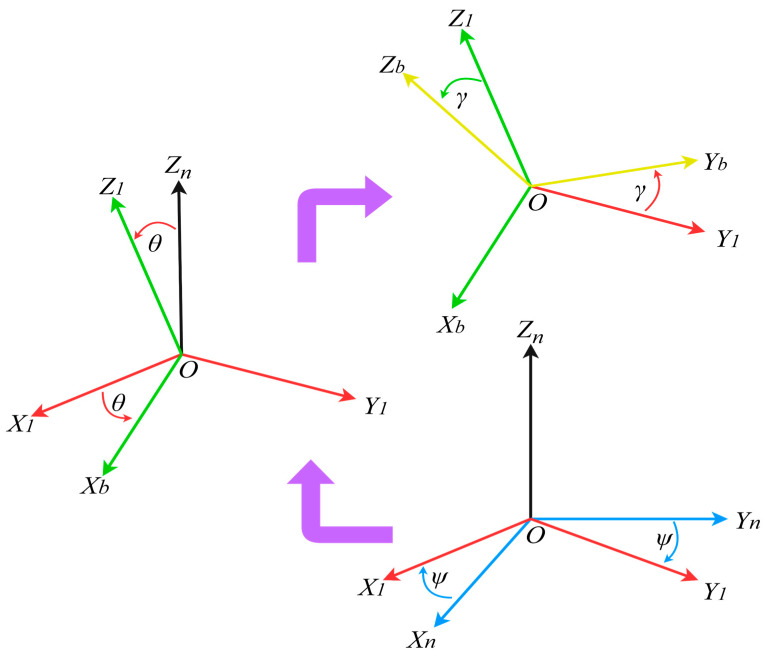
Conversion of n coordinate system to b coordinate system.

In the IMU system, usually in order to explain the robot’s position, movement speed, direction, etc., the corresponding coordinate system must be selected. In this paper, we use the IMU’s installation plane as the carrier coordinate system (b coordinate system). Additionally, we select the geographical coordinate system as the navigation coordinate system (n coordinate system) to simplify the attitude calculation and improve comprehension [19]. The three axes (*X*, *Y*, *Z*) of the n coordinate system are commonly achieved by sequentially rotating the angles of roll (*ψ*), pitch (*γ*), and yaw (*θ*). The conversion process is shown in Figure 1.

The transformation matrix between the n coordinate system and the b coordinate system is often referred to as the direction cosine matrix. The coordinate transformation of the direction cosine matrix can be expressed as follows:

(1) $$\left[\begin{array}{c} x_{b}\\ y_{b}\\ z_{b} \end{array}\right]=C_{n}^{b}\left[\begin{array}{c} x_{n}\\ y_{n}\\ z_{n} \end{array}\right]$$

In this formula, the subscript b represents the carrier coordinate system, the subscript n represents the navigation coordinate system, and $C_{n}^{b}$ represents the rotation matrix from the carrier coordinate system to the navigation coordinate system. This can be expressed using Equation (2).

(2) $$\begin{array}{c} C_{n}^{b}=\left[\begin{array}{ccc} \cos\theta \cos\psi & \cos\theta \sin\psi & -\sin\theta \\ \sin\gamma \sin\theta \cos\psi -\cos\gamma \sin\psi & \cos\gamma \cos\psi +\sin\gamma \sin\theta \sin\psi & \sin\gamma \cos\theta \\ \sin\gamma \sin\psi +\cos\gamma \sin\theta \sin\psi & \cos\psi sin\gamma \cos\psi -\cos\gamma \sin\theta & \cos\gamma \cos\theta \end{array}\right] \end{array}$$

This representation method has a flaw known as gimbal lock, which can result in the loss of one posture’s degree of freedom. To avoid this issue and provide a more concise description of the robot’s motion, the four-element method [20] is commonly used. This method is similar to algebraic complex numbers but is more compact and free of singularities. Let the rotational quaternion be q, whose common representation is shown in Equation (3).

(3) $$\begin{array}{c} q=q_{0}+q_{1}i+q_{2}j+q_{3}k \end{array}$$

where $q_{0},\;q_{1},q_{2},q_{3}$ are i, j,k real numbers and are the other three imaginary parts of the quaternion.

The quaternionic representation of the rotation matrix is shown in Equation (4).


(4) $$\begin{array}{c} C_{n}^{b}\left(q\right)= \end{array}\left[\begin{array}{ccc} q_{0}{}^{2}+q_{1}{}^{2}-q_{2}{}^{2}-q_{3}{}^{2} & 2\left(q_{0}q_{3}+q_{1}q_{2}\right) & 2\left(q_{1}q_{3}-q_{0}q_{2}\right)\\ 2\left(q_{1}q_{2}-q_{0}q_{3}\right) & q_{0}{}^{2}-q_{1}{}^{2}+q_{2}{}^{2}-q_{3}{}^{2} & 2\left(q_{2}q_{3}+q_{0}q_{1}\right)\\ 2\left(q_{1}q_{3}+q_{0}q_{2}\right) & 2\left(q_{2}q_{3}-q_{0}q_{1}\right) & q_{0}{}^{2}-q_{1}{}^{2}-q_{2}{}^{2}+q_{3}{}^{2} \end{array}\right]$$

By jointly solving Equations (2) and (4), the quaternion representations of *θ*, *γ*, and *ψ* can be obtained, as shown in Equations (5)–(7).

(5) $$\begin{array}{c} \psi =\mathrm{atan}2\left(2\left(q_{1}q_{2}+q_{0}q_{3}\right),\left(q_{0}^{2}+q_{1}^{2}-q_{2}^{2}-q_{3}^{2}\right)\right) \end{array}$$

(6) $$\begin{array}{c} \theta =\arcsin\left(-2\left(q_{1}q_{3}-q_{0}q_{2}\right)\right) \end{array}$$

(7) $$\begin{array}{c} \gamma =\mathrm{atan}2\left(2\left(q_{2}q_{3}+q_{0}q_{1}\right),\left(q_{0}^{2}-q_{1}^{2}-q_{2}^{2}+q_{3}^{2}\right)\right) \end{array}$$

The IMU has both advantages and disadvantages. In an IMU, both accelerometers and gyroscopes use an integral method to measure data. For example, integrating linear acceleration can obtain linear velocity, and integrating again can obtain position information. The rotation angle can also be obtained by integrating the angular speed measured using the gyroscope. However, when attempting to solve the state quantity through integration, particularly in discrete form, it is inevitable that various errors will be introduced. The IMU errors are mainly divided into systematic errors and random errors. The former mainly include the scale factor error, axis deviation error, bias error, and temperature error, while the latter mainly include random walk and bias instability. Systematic errors usually follow certain patterns, which can be eliminated through real-time compensation. Random errors, on the other hand, typically refer to noise, and it is often challenging to identify a suitable function to describe the noise, making the processing quite complex. Usually, the manufacturer corrects the systematic errors in the IMU chip when leaving the factory [21]. As for random errors, various data fusion technologies have been developed to overcome them [22,23,24].

Random walk and bias instability are key parameters in the fused positioning system. There are related calculations in many fused SLAM algorithms involving the IMU (such as VINS and ORB-SLAM3). Once the above parameters are obtained, a covariance is set on the pre-integrated error term of the IMU [25], which in turn reduces the impact of random errors on the overall system. This article uses the Allan variance [26,27] method (this method is one of the time series analysis methods) to conduct error modeling analysis on the zero-point offset data. By processing the MEMS inertial device output data using the Allan variance method, various error coefficients can be identified more accurately.

The steps of the Allan variance calculation are as follows.

1. Acquire and group the data.

Set a fixed sampling frequency f, collect the output value of the inertial device, collect a total of N sampling points, and obtain the sample space. Divide each n data point into M independent arrays, which can be expressed by Equation (8).

(8) $$\begin{array}{c} M=\left(\frac{N}{n}\right) \end{array}$$

2. Calculate the average value of the data.

Calculate the average value of each set of raw output data, i.e., the average value of the dataset ${\bar{\omega }}_{k}\left(n\right)$, to obtain an array of random variables as shown in Equation (9).


(9) $${\bar{\omega }}_{k}\left(n\right)=\frac{1}{n}\overset{n}{\underset{i=1}{\sum }}\omega_{\left(k-1\right)n+i},\;k=1,2,\cdots ,M$$

3. Obtain the Allan variance.

Defining the sampling time of each group as $\tau_{n}=n/f$, as a time series, the Allan variance is defined as Equation (10).


(10) $$\sigma^{2}\left(\tau_{n}\right)=\frac{1}{2\left(M-1\right)}\overset{M-1}{\underset{k=1}{\sum }}{\left({\bar{\omega }}_{k+1}{\left(n\right)}^{2}-{\bar{\omega }}_{k}\left(n\right)\right)}^{2}$$

**Figure 2:**
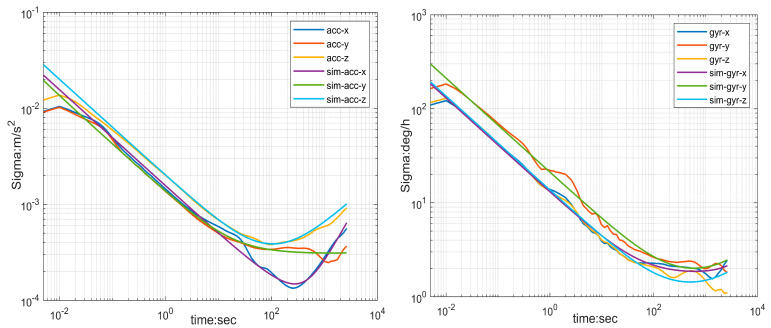
Allan variance curve of accelerometer (**left**) and gyroscope (**right**) over time. sim-acc and sim-gyr are the data output from the analog accelerometer and analog gyroscope in imu_untils, respectively.

In this paper, data were collected by keeping the IMU stationary for about 2.5 h, using a frequency of 200 Hz. The output data were processed according to the Allan variance formula described above. The Allan variance curve is shown in Figure 2 and the obtained error parameters are shown in Table 2. The specific analyzing tools used are Kalibr toolbox [28] and imu_utils [29].

### 2.2. IMU Fusion Method Based on Extended Kalman Filter

Because the Kalman Filter (KF) is more suitable for linear systems, and our system has many sensors, there are many uncertainties, so we cannot continue to use this algorithm. The EKF applies linear analysis to the KF model, allowing for the use of a nonlinear model in the prediction and update process of the linear model. Suppose there is a robot that measures information such as linear velocity through a wheel odometry and uses an IMU to measure information such as acceleration and angular velocity. This paper presents a new odometry that utilizes the EKF for data fusion from two sensors, enabling accurate estimation of the robot’s attitude and position. The EKF divides the system into prediction and update steps, which are implemented based on the system’s motion model and observation model, respectively [30,31].

The state of the robot is represented by the state vector, and the state equation is shown in Equation (11).

(11) $$\begin{array}{c} x_{k}={\left[p_{x},p_{y},p_{z},\theta ,\gamma ,\psi ,v_{x},v_{y},v_{z},b_{a},b_{g}\right]}^{⊤} \end{array}$$

where $p_{x},p_{y},p_{z}$ are the positions of the robot in the world coordinate system. *θ*, *γ*, *ψ* are the Euler angles of the robot. $v_{x},v_{y},v_{z}$ are the velocities of the robot in the world coordinate system, and $b_{a},b_{g}$ are the bias of the accelerometer and gyroscope of the IMU, respectively.

The motion of the robot is described by a nonlinear system model, as shown in Equation (12).

(12) $$\begin{array}{c} x_{k}=f\left(x_{k-1},u_{k-1},\Delta t\right)+w_{k-1} \end{array}$$

where $f\left(x\right)$ is the state transition function, $u_{k-1}$ is the IMU measurement value, Δt is the time interval, and $w_{k-1}$ is the process noise.

The observation equation of the robot is shown in Equation (13).

(13) $$\begin{array}{c} z_{k}=h\left(x_{k}\right)+v_{k} \end{array}$$

where $h\left(x_{k}\right)$ is the observation matrix that maps the robot’s state to the output space of the IMU and odometry, and $v_{k}$ is the observation noise.

Finally, the prediction state and the covariance matrix are divided into the following four steps:

1. Define the prior estimate, as shown in Equation (14).
  (14) $${\hat{x}}_{k}=f\left({\hat{x}}_{k-1},u_{k},0\right)$$
  where ${\hat{x}}_{k-1}$ represents the posterior estimate at the moment k−1.
2. Define the prior estimation error covariance matrix, as shown in Equation (15).
  (15) $$P_{k}^{-}=F_{k-1}P_{k-1}^{-}F_{k-1}^{T}+Q_{k}$$
  where $F_{k-1}=\frac{\partial f}{\partial x}{|}_{{\hat{x}}_{k-2}}$ is the Jacobian matrix of the state transition matrix, and $Q_{k}$ is the covariance matrix of the process noise.
3. Define posterior estimation as shown in Equation (16).
  (16) $${\hat{x}}_{k}={\hat{x}}_{k-1}+K_{k}\left(z_{k}-h\left({\hat{x}}_{k-1},0\right)\right)$$
  where $K_{k}$ is the Kalman gain.
  (17) $$K_{k}=P_{k}^{-}H_{k}^{T}{\left(H_{k}P_{k-1}^{-}H_{k}^{T}+R_{k}\right)}^{-1}$$
  where $P_{k}^{-}$ represents the covariance matrix of the prior estimation error, $H_{k}=\frac{\partial h}{\partial x}{|}_{{\hat{x}}_{k-1}}$ is the Jacobian matrix of the observation matrix, and $R_{k}$ is the covariance matrix of the observation noise.
4. Define the posterior estimation error covariance matrix, as shown in Equation (18).
  (18) $$P_{k}=\left(I-K_{k}H_{k}\right)P_{k-1}^{-}$$
  where *I* is the identity matrix.

From the calculation process of the above formula, it is clear that the EKF can effectively incorporate the conducive information in the observation value $z_{k}-h\left({\hat{x}}_{k-1},0\right)$ through the Kalman gain ($K_{k})$. This, in turn, enables the algorithm to correct the prior estimate of the state vector by giving more weight to the useful information. This paper utilizes state estimation from the IMU and wheel odometry for prediction, and the positioning result from RTABMAP as the observation to update the state vector [32]. In this paper, the ROS function package robot_localization [33] is used to realize the fusion location of the EKF.

### 2.3. RTABMAP Improvement Method

The IMU is not affected by the environment when solving poses. Therefore, it can estimate the speed, position, and attitude of all parameters solely based on the inertial information generated by the carrier’s motion. The improvement method in this paper introduces the IMU for fusion in the framework of the RTABMAP algorithm based on visual-wheel-odometry. The IMU can estimate motion parameters that can be utilized to rectify the absence of scale information in the camera. It can also offer high-frequency attitude estimation data (acceleration and angular velocity) and real-time rapid response positioning [34]. On the other hand, the wheel odometry can obtain a more precise displacement and linear speed compared to the IMU. Furthermore, the camera’s measurement of the carrier motion can rectify the cumulative error of the IMU. The IMU provides absolute proportions and accurate poses in a short period of time at a much faster sampling rate than images [35]. Integrating the camera with the IMU and wheel odometry can enhance the robustness and positioning accuracy of the algorithm. This integration can also improve the inaccurate results of the visual algorithm caused by certain movements or scenes with simple textures [36].

**Figure 3:**
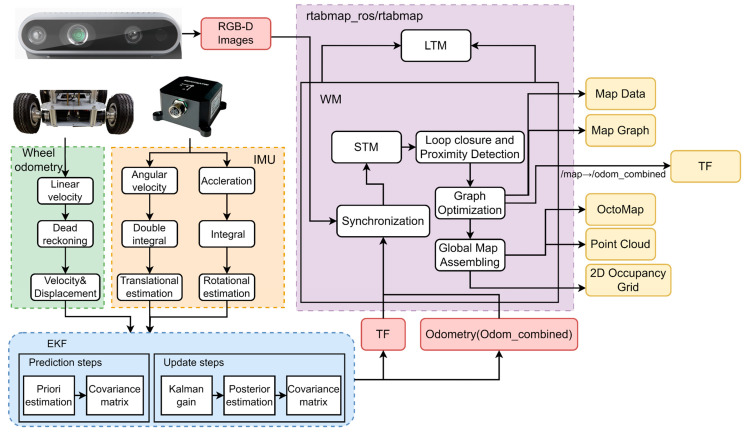
Improved RTABMAP system architecture TF is a package that lets the user keep track of multiple coordinate frames over time.

The proposed system has two main parts: multi-sensor fusion localization and 3D dense point cloud mapping. The multi-sensor fusion localization section integrates position estimation from wheel odometry, the IMU, and cameras. The robot obtains displacement and velocity through trajectory extrapolation with the help of wheel odometry, and at the same time obtains angular velocity, angular displacement, and acceleration with the integration algorithms of the IMU’s gyroscopes and accelerometers. Finally, the EKF is utilized to fuse the above data into a motion model of the robot, and the robot’s state is updated in real-time by the prediction step and update step of the EKF. Due to the different locations of the sensors in space, there is also a translation of the coordinate system between the sensors, so the EKF will eventually provide a TF coordinate translation as well as an odometry called Odom_combined, which is then time-synchronized with the camera’s raw data and deposited into the node created by the STM. The node also holds visual words for loop closure and similarity detection (similar to the visual bag-of-words model of ORB-SLAM2) and a local occupation grid for building a global map. Finally, the corresponding solution is performed based on the data in the node, and the solution result will be used as the constraints for the graph optimization (next node). When there is a new loop closure or proximity link added to the graph, graph optimization propagates computational errors throughout the map to reduce odometry drift and performs ranging corrections via TF transformation to correct the robot’s localization in the map. For the 3D dense point cloud mapping, the original RTABMAP method is used to generate a real-time dense three-dimensional point cloud map of the robot’s environment in rtabmapviz. The specific system framework is shown in Figure 3.

## 3. Experiment

To validate the improved method’s effectiveness in real-world scenarios, this paper assesses localization accuracy through two dataset experiments and three real-world experiments. The dataset is the V1 series in EuRoC, the real experimental scenarios are all indoors, and each experimental site is of different sizes and contains complex environmental conditions to verify the feasibility under different settings. In this paper, the original method is still called RTABMAP. The improved method is called RTABMAP-VIWO, abbreviated as VIWO. We use the EVO [37] to compare our experimental results. This tool enables users to visualize, analyze, and statistically evaluate SLAM algorithms and trajectory data from public datasets. It helps to make the evaluation process more intuitive and informative. The assessment indicator is the absolute pose error (APE) value that considers rotation and rotation. APE is a measurement utilized for examining the overall coherence of trajectories in SLAM. APE is based on the absolute relative pose between two poses $P_{ref,i},P_{est,i}\in \mathrm{SE}\left(3\right)$ at timestamp i.

(19) $$E_{i}=P_{est,i}⊖P_{ref,i}=P_{ref,i}^{-1}P_{est,i}\in \mathrm{SE}\left(3\right)$$

where ⊖ represents the inverse compositional operator that calculates the relative poses between two given poses. $P_{est,i}$ and $P_{ref,i}$ are the estimated and true postures, respectively.

In the calculation of APE, the translation error ($APE_{trans,i}$) and rotation error ($APE_{rot,i}$) are calculated, respectively, by using the translation part and rotation part of $E_{i}$.

(20) $$APE_{trans,i}=∥\mathrm{trans}\left(E_{i}\right)∥$$

(21) $$APE_{rot,i}=∥\mathrm{rot}\left(E_{i}\right)-I_{3\times 3}{∥}_{F}$$

where $I_{3\times 3}$ is the identity matrix.

The complete error taking into account rotation and translation errors is calculated using the full $E_{i}$.

(22) $$APE_{i}=∥E_{i}-I_{4\times 4}{∥}_{F}$$

where $I_{4\times 4}$ is the identity matrix.

Finally, for ease of understanding, this paper uses the Root-Mean-Square Error (RMSE) to evaluate the accuracy, which is calculated as shown in Equation (23).

(23) $$RMSE=\sqrt{\frac{1}{N}\sum_{i=1}^{N}APE_{i}^{2}}$$

where *N* represents the Nth timestamp.

In real-world scenarios, we use RGB-D cameras as the primary sensor, but for dataset testing purposes, we rely on stereo cameras as the primary sensor due to the types of sensor limitations of the EuRoC dataset. Despite the difference in sensors, we are still able to validate the feasibility of our proposed method.

### 3.1. Evaluation and Analysis of EuRoC Dataset

The dataset utilized in this article is the EuRoC MAV visual inertial dataset [38], which was gathered using the AscTec “Firefly” hex-rotor helicopter. The data were collected through a binocular camera (AptinaMT9V034, 20 Hz) and a synchronous measurement MEMSIMU (ADIS16448, 200 Hz), along with the Vicon motion capture system (VICON) and laser tracker (LeicaMS50). This part of the experiment only involves the stereo camera and IMU, and the other sensors can be ignored. RTABMAP is a visual odometry (stereo camera odometry) and VIWO is a visual-imu odometry (stereo camera + IMU odometry). The hardware environment for running the dataset is a laptop with the following specifications: CPU: i5-7300HQ, 2.50 GHz × 4; RAM: 16 GB; GPU: NVIDIA GTX1050Ti; operating system: Ubuntu 18.04; ROS: Melodic.

#### 3.1.1. Running in Dataset V1_01_easy

**Figure 4:**
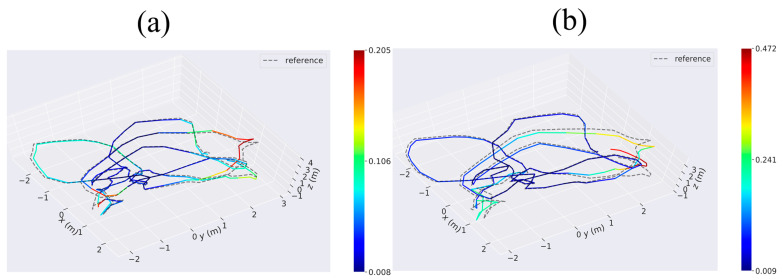
The absolute pose error (APE) in V1_01_easy considers both rotation and translation errors. Column (**a**): absolute pose error of VIWO. Column (**b**): absolute pose error of RTABMAP. reference: actual trajectories of the robot.

**Figure 5:**
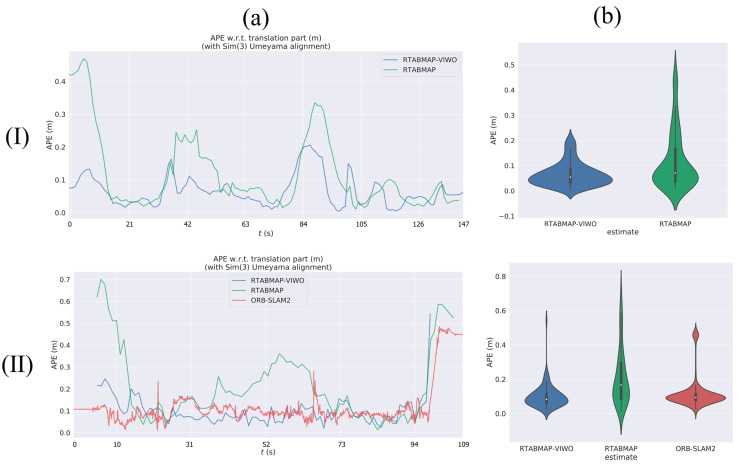
Comparison of APE values (RES) between VIWO, RTABMAP, and ORB-SLAM2 in V1_01_easy and V1_03_difficult. Column (**a**) is a comparison of multiple absolute pose error (APE) changes over time. Column (**b**) is a comparison of violin plots. Row (**I**): V1_01_easy, Row (**II**): V1_03_difficult.

For this experiment, we use the V1_01_easy sequence (V101) to assess and compare the trajectories of VIWO and RTABMAP. To ensure a fair and convenient comparison, we compare VIWO with the original method using the actual trajectories from the dataset, and compare one or more APEs using the evo_res tool in EVO. In the V101 sequence, the camera direction remains constant regardless of the drone’s attitude, resulting in minimal stimulation of the three-axis gyroscope in the IMU. The absolute pose error map of the V101 sequence is shown in Figure 4a,b, it can be seen that the errors of our method in several large turns are small RTABMAPs, and the maximum error is only 43% of RTABMAP. A comparison of violin plots between the two methods is shown in Figure 5b(I). The results show that the APE of VIWO is obviously reduced in this sequence. As a result, only the translation error can be compared in this sequence, while the rotation error can be disregarded. Table 3 displays the error parameters for the two evaluation methods. Upon comparative analysis, it is evident that the RMSE decreases by 48.1% and the standard error decreases by 57.8%. As a result, the trajectory estimated by VIWO is more accurate and closely resembles the actual trajectory. Although the improvement in RTABMAP-VIWO in V101 is not significant, it still verifies the feasibility.

#### 3.1.2. Running in Dataset V1_03_difficult

In the V101 sequence, this paper demonstrates the feasibility of making improvements to RTABMAP, and the performance enhancement effect of VIWO is further demonstrated subsequently. In this test, we conduct comparison experiments using the V1_03_difficult sequence (V103) and include ORB-SLAM2 in the comparison. Both the RTABMAP and ORB-SLAM2 methods utilize feature extraction to extract and match feature points in the image. Both methods are based on nonlinear optimization to optimize the camera position and map, and both include complete loop closure detection and support multiple cameras. Therefore, they share many similarities in the algorithm’s details. Because ORB-SLAM2 produces sparse point cloud maps, its performance is superior. During the experiment, the trajectory of ORB-SLAM2 appears smoother due to its recording of more pose keyframes compared to RTABMAP. 

**Figure 6:**
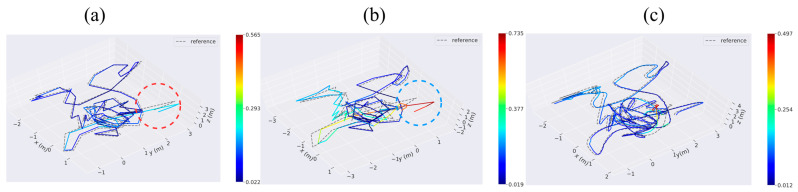
The absolute pose error (APE) in V1_03_difficult considers both rotation and translation errors. Column (**a**): absolute pose error of VIWO. Column (**b**): absolute pose error of RTABMAP. Column (**c**): absolute pose error of ORB-SLAM2. Reference: actual trajectories of the robot.

The V103 sequence was recorded in a challenging environment, ranging from slow flight with good visibility to dynamic flight with motion blur and poor lighting. This posed a significant increase in difficulty compared to the V101 sequence. Upon comparing the blue mark ring in Figure 6a to the red mark ring in Figure 6b, it becomes apparent that near this position (the time node is located between 103 s and 109 s of V103 sequence), RTABMAP experiences a significant trajectory drift, whereas VIWO, under the constraint of the IMU, aligns more closely with the actual trajectory. Labbé [18] shows that in V1 sequences, the performance of RTABMAP is worse than that of ORB-SLAM2 because the global bundle adjustment performed by ORB-SLAM2 on loop closures would then give better optimization than only using graph optimization performed by RTABMAO for these sequences. However, in this paper, we use the EKF to fuse IMU data, which significantly improves the performance of VIWO. As shown in Table 4, the RMSE is reduced by 59.2%, and the standard error is reduced by 60.3%. A comparison of violin plots between the three methods is shown in Figure 5b(II), and it is clear from the figure that our method has a lower median, as well as a lower discrete stratification of the APE. Moreover, the difference between the results of other evaluations and those of ORB-SLAM2 is negligible, and in some cases, our results even outperform those of ORB-SLAM2.

### 3.2. Real Environment Verification and Analysis

**Figure 7:**
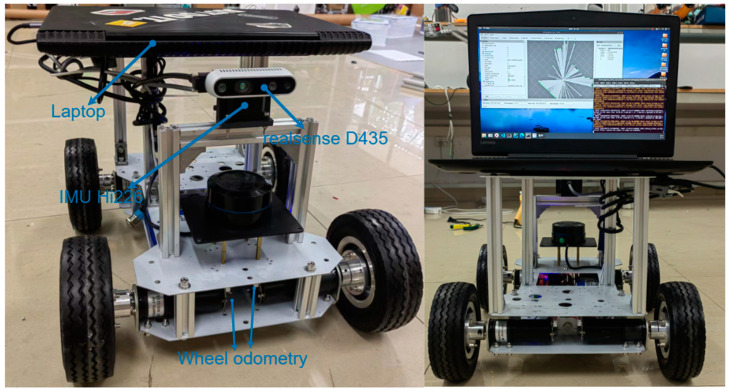
Wheeled mobile robot for experiment.

In order to better validate the robustness and accuracy of the positional trajectories of RTABMAP-VIWO in the real world, we built a wheeled mobile robot driven by four DC reduction motors with encoders. The motor operating voltage is 24 V, and the steering method is differential steering. The controller chip of the lower system is STM32F407VET6, and the controller used by the upper system is the same laptop that was used to run the dataset, CPU: i5-7300HQ, 2.50 GHz × 4; RAM: 16 GB; GPU: NVIDIA GTX1050Ti; camera: realsenseD435; RGB FOV (H × V): 69° × 42°; fps: 30; IMU: HiPNUC Hi226 (6-DOF); refresh rate: 200 Hz; operating system: Ubuntu 18.04; ROS: Melodic. The mounting position of each sensor is shown in Figure 7.

**Figure 8:**
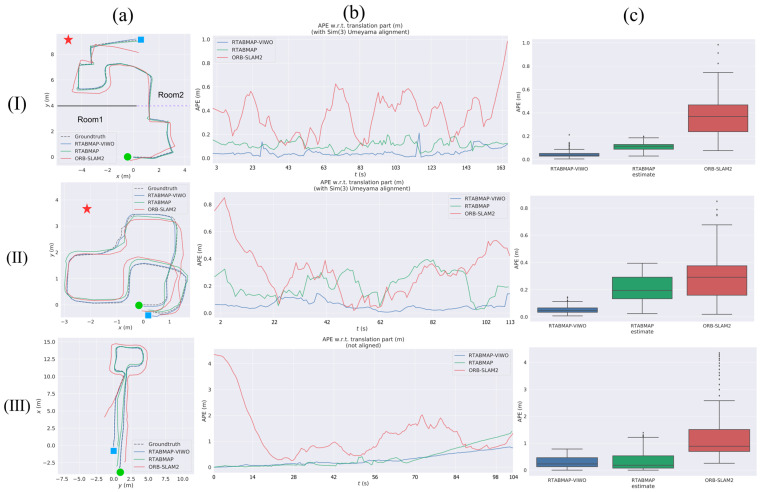
Comparison of APE values (RES) between VIWO, RTABMAP, and ORB-SLAM2 from test 1 to test 3. Column (**a**) is a comparison of the absolute positioning errors of the three methods. Column (**b**) is a comparison of multiple APE changes over time. Column (**c**) is a comparison of box plots. Groundtruth: actual trajectories of the robot. Row (**I**): test 1, Row (**II**): test 2, Row (**III**): test 3. In column (**a**), the green circle is the start of the experimental route, the blue rectangle is the end of the route, and the red pentagram is the location of the air conditioner. In column (**a**) of Row (**I**), the black line is the wall and the purple dotted line is the connecting passages.

Figure 8 shows the RES of VIWO, RTABMAP, and ORB-SLAM2 in test 1 to test 3, respectively. The quantitative results comparison report is shown in Table 5. In real-world testing, RTABMAP is a visual-wheel odometry (RGB-D camera + Wheel odometry), VIWO is a visual-imu-wheel odometry (RGB-D camera + IMU + Wheel odometry), and ORB-SLAM2 is a visual odometry (only RGB-D camera).

#### 3.2.1. Runing in Closed Room Scenario 1

**Figure 9:**
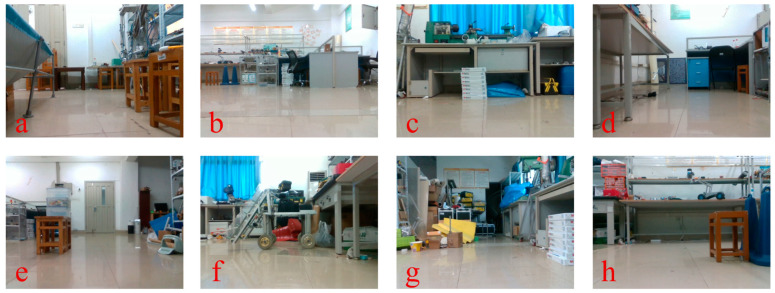
Some images captured and used for camera computation in test 1 (**a**–**d**) and test 2 (**e**–**h**).

During test 1, the mobile robot navigated an S-shaped path indoors. The experiment involved two methods of turning: turning on the spot and turning at a differential speed. Scenario 1 is a large, connected laboratory (approximately 12 m × 11 m in size) consisting of Room 1 and Room 2, where Room 1 has no air conditioning vibrations and Room 2 has air conditioning vibrations, and the rest of the conditions are the same in both rooms. The dividing walls and connecting passages of the two rooms are shown in Figure 8a(I). The environment included strong reflective lights on the ground and the vibration from the air-conditioning. The robot moves from Room 1 to Room 2, so the first half of the journey is a little affected by the air-conditioning vibrations, and in the second half of the journey, the closer you are to the air-conditioning, the more the vibrations increase. Real-life scenarios are depicted in Figure 9, as seen in images a through d.

**Figure 10:**
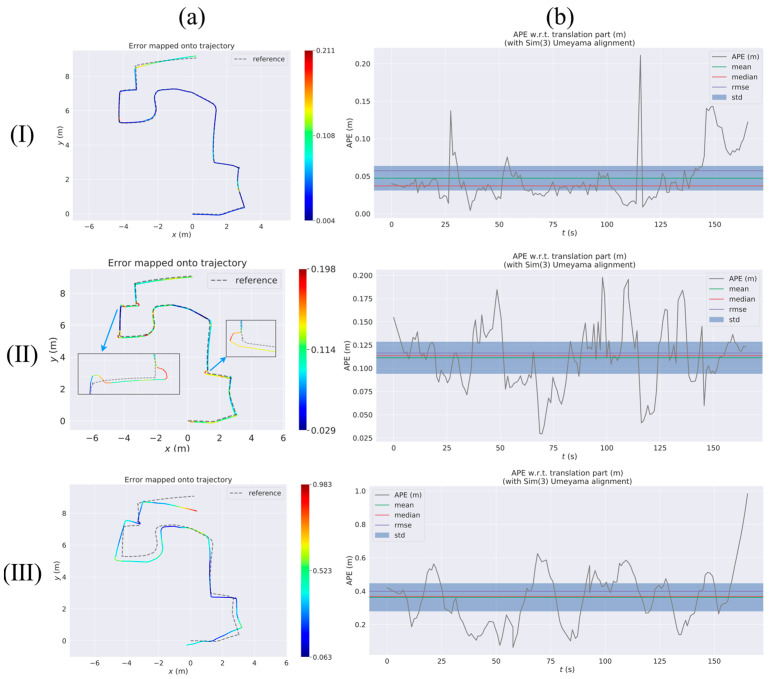
Absolute pose error (APE) of test 1 considering both rotation and translation errors. Row (**I**): RTABMAP-VIWO, Row (**II**): RTABMAP, Row (**III**): ORB-SLAM2. Column (**a**) shows the error trajectory of the three methods, while Column (**b**) displays the curve of APE changing over time for each method. reference: actual trajectories of the robot.

Figure 10(II) shows that RTABMAP’s visual wheel odometry estimation is relatively accurate. However, when using the turn-in-place approach to navigate a right-angle corner, the estimated trajectory drifts and the error increases linearly. This contrasts with the performance of VIWO in Figure 10(I). By examining test 1 of Table 5, it is evident that although VIWO has a maximum absolute position error of 0.211, its mean error (Mean) is significantly lower than that of RTABMAP. This indicates that the trajectory estimated by VIWO is closer to the actual trajectory. Additionally, the APE curves of our proposed method are much smoother, demonstrating the higher robustness of our system. Additionally, the overall performance of ORB-SLAM2 in test 1 is inferior to that of the dataset. Upon analyzing the error map of ORB-SLAM2, we discovered that the trajectory error increases as the robot approaches the placement of the air conditioner. A comparison of the trajectories of the three methods is shown in Figure 8a(I). The experimental results show that the error assessment index of our proposed method is significantly lower than the error value of RTABMAP with ORB-SLAM2, and VIWO is highly suitable for positioning robots in challenging environments.

#### 3.2.2. Runing in Closed Room Scenario 2

Mobile robots are typically assigned closed-loop routes to travel, regardless of the scenario they are used in. Hence, in test 2, this paper assesses the VIWO via a closed-loop pathway. The entirety of test 2 in Scenario 2 takes place in Room 2, so the environment in test 2 encompasses light reflection and air-conditioner vibration. Refer to Section 3.2.1 for the difference between Room 1 and Room 2. And a portion of the actual scene is depicted in images e through h in Figure 9. 

**Figure 11:**
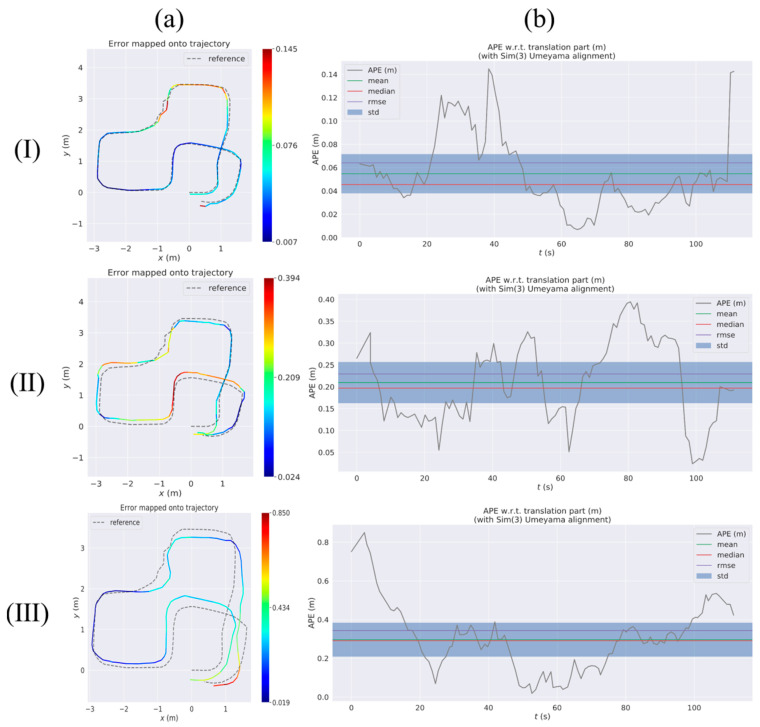
Absolute pose error (APE) of test 2 considering both rotation and translation errors. Row (**I**): RTABMAP-VIWO, Row (**II**): RTABMAP, Row (**III**): ORB-SLAM2. Column (**a**) shows the error trajectory of the three methods, while column (**b**) displays the curve of APE changing over time for each method. Reference: actual trajectories of the robot.

As shown in Figure 11a(I), the VIWO proposed in this paper achieves a more accurate closed-loop effect, while the RTABMAP exhibits translational errors at both the bend and end points. The performance of ORB-SLAM2 is similar to that in test 1, where the estimated trajectories drift due to light reflections from the ground and air-conditioning noise. By examining test 2 of Table 5, it is evident that our proposed VIWO has an RMSE of 0.064. Additionally, the mean absolute error (Mean) of VIWO is only one-fourth that of RTABMAP and one-sixth that of ORB-SLAM2. These values are lower than the corresponding values for RTABMAP and ORB-SLAM2. In addition, other error coefficients due to our proposed method are lower than those of the other two methods. A comparison of box plots between the three methods is shown in Figure 8c. Hence, the experimental outcomes of test 1 and test 2 demonstrate that VIWO can offer not only a precise positioning estimation outcome but also facilitate the navigation of mobile robots in closed-loop paths in challenging surroundings.

#### 3.2.3. Testing in Indoor Corridors

**Figure 12:**
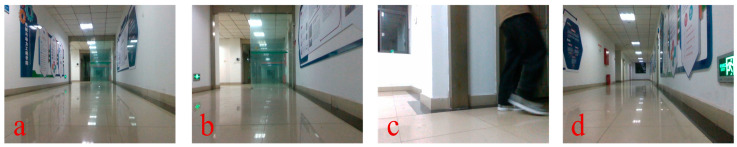
Some of the live images captured by the camera in test 3. (**c**) is passing pedestrians. (**a**,**b**,**d**) are actual views of the corridor.

In indoor settings, it is inevitable to encounter long, narrow corridors or tunnel-like structures with a high degree of repetitive features. In test 3, we test the mobile robot in this scene. As shown in Figure 12, this scene has unique characteristics that include stronger light illumination, ground light reflection, and walls with high similarity compared to test 1 and test 2. Additionally, there are two transparent glass doors and passing pedestrians in the scene. These factors will pose significant challenges to the precise positioning of the mobile robot.

**Figure 13:**
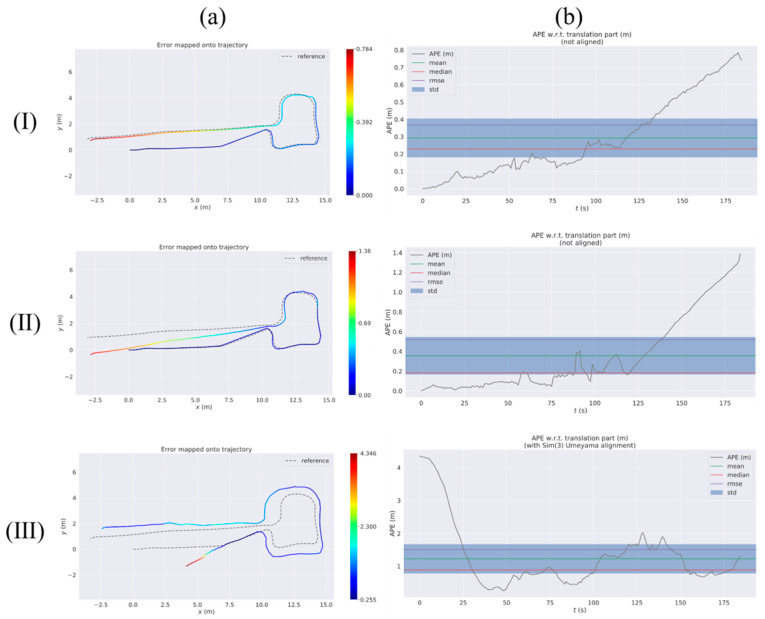
Absolute pose error (APE) of test 3 considering both rotation and translation errors. (**I**): RTABMAP-VIWO, (**II**): RTABMAP, (**III**): ORB-SLAM2. Column (**a**) shows the error trajectory of the three methods, while column (**b**) displays the curve of APE changing over time for each method. reference: actual trajectories of the robot.

As shown in Figure 13a(II), the localization accuracy of RTABMAP during the latter part of the driving journey is unsatisfactory. This is due to the repetitive nature of the environment, which has few feature points and results in a noticeable trajectory drift. RTABMAP, even with the aid of the wheel odometry, performs only moderately well in this scenario. On the other hand, in harsh environmental conditions, ORB-SLAM2 experienced a more severe trajectory drift compared to test 1 and test 2. The trajectory was even deflected to a 3D trajectory due to light reflection, with the APE (Max) reaching an astonishing 4.34 and the RMSE about 1.51, much higher than those of the other two methods. Specific results are shown in test 3 of Table 5. During the actual experiments, both RTABMAP and ORB-SLAM2 experienced a certain number of estimation failures. On the contrary, our proposed method in this paper significantly improves the positioning accuracy by using the EKF loosely coupled wheel odometry and IMU for prediction, as shown in Figure 13(I). The RMSE of VIWO is 0.36, while the RMSE of RTABMAP is about 0.52, as shown in test 3 in Table 5. Hence, the method proposed in this paper effectively minimizes the localization error and misjudgment rate of RTABMAP in intricate corridor settings. Additionally, it further validates the practicability of multi-sensor fusion techniques in achieving more precise positioning in complex settings.

## 4. Results and Discussion

The results of the dataset experiments are shown in Table 3 and Table 4, and the RMSE of our proposed method is reduced by 48.1% and 59.2% compared to the RTABMAP. The author of RTABMAP [18] mentioned ORB-SLAM2 in the original paper and performed comparison experiments, and the results show that ORB-SLAM2 performs better than RTABMAP in this dataset. And the RMSE coefficient of our proposed method is 24.2% lower compared with ORB-SLAM2. The results of the real environment experiments are shown in Table 5. In the three experiments in the real environment, the RMSE of our proposed method is reduced by 50.8% and 72.0% compared with the original method, and even in test 3, which is the harshest environment, the RMSE of our proposed method is reduced by 29.1%. ORB-SLAM2 does not perform well in real environments, due to environmental factors, and its performance is much worse than that of our proposed method and the RTABMAP. 

In this paper, we conduct experiments on the EuRoC dataset with the main purpose of verifying the feasibility of our method. However, the dataset experiments are different from the real environment, and the purpose of our improvement is to enhance the performance of RTABMAP and to be able to be applied in real life, so we conducted the experiments in a real environment with multiple common interferences. Five experiments ranging from simple to difficult finally proved that the RTABMAP-VIWO system utilizing the EKF fusion IMU is superior to RTABMAP and exhibits higher positioning accuracy and anti-jamming.

There are limitations that exist in our method. The individual sensors in the wheeled robot we built are mainly fixed by aluminum as well as 3D-printed non-standard parts, which creates a considerable mounting error for the TF conversion in the whole system. In addition, when operating in the environment shown in Figure 12, the robot can experience a large trajectory drift if the presence of liquid on the ground causes the wheels to slip.

## 5. Conclusions

Aiming at the complex indoor environment, this paper proposes an improved RTABMAP method. To achieve precise and reliable robot pose estimation in indoor environments, this paper integrates the IMU into the original system using the EKF to minimize positioning errors caused by the primary sensor. By combining the 3D dense point cloud map, constructed using RTABMAP, with the proposed visual inertial wheel odometry estimation, mobile robots can achieve more accurate indoor repositioning. This provides more precise sensory information for further path planning and autonomous navigation.

This paper conducts tests and evaluations in both common and challenging indoor environments. The performance of three SLAM methods is compared in both public datasets and real-world scenarios. In RTABMAP, motion estimation during localization or loop closure detection is mainly performed visually. The experimental results demonstrate that both RTABMAP-VIWO systems, using stereo and RGB-D cameras as the primary sensors, respectively, exhibit low drift error and high robustness. In addition, the main work of this paper is the improvement in the localization accuracy of the mobile robot, while the map construction and navigation have not been studied in depth. In the future, we will incorporate low-cost 2D lidar, which will improve the robot’s localization accuracy and build a higher-quality map, creating the basis for subsequent robot navigation. In addition, we will combine SLAM with deep learning to further reduce the impact of reflective ground lighting and passing pedestrians on localization.

## Figures and Tables

**Table 1:** Comparison of the SLAM methods and supported inputs and outputs.

	Inputs							Outputs			
Method	Camera			IMU	Lidar		Wheel	Pose	Occupancy		Point Cloud
	Mono	Stereo	RGB-D		2D	3D			2D	3D	
VINS-Mono	✓			✓				✓			Sparse
ORB-SLAM2	✓	✓	✓								Sparse
RTABMAP	✓	✓	✓		✓	✓	✓	✓	✓	✓	Dense
DM-VIO	✓			✓				✓			Dense
VIWO	✓	✓	✓	✓	✓	✓	✓	✓	✓	✓	Dense

VIWO: RTABMAP-VIWO, Mono: monocular, Wheel: wheel odometry.

**Table 2:** Error parameters and their corresponding values.

Variables	Meanings
Sampling interval of IMU sensor	200 Hz
Gyroscope measurement noise variance, $\sigma_{g}$	0.00109 rad/s
Accelerometer measurement noise variance, $\sigma_{a}$	0.0234 rad/s
Gyroscope measurement “bias Instability”, $\sigma_{bg}$	0.00000859 m/s^2^
Accelerometer measurement “bias Instability”, $\sigma_{ba}$	0.000697 m/s^2^

**Table 3:** Comparisons between our proposed method and RTABMAP were made based on the APE values.

Dataset ID	Method	Std	RMSE	Min	Median	Mean	Max
V101	RTABMAP-VIWO	0.04894	0.08539	0.00757	0.06353	0.06997	0.20492
	RTABMAP	0.11488	0.16415	0.01657	0.06311	0.11725	0.47280

Std: standard error, RMSE: Root-Mean-Square Error, Min: minimum error, Median: median error, Mean: mean absolute error, and Max: maximum error.

**Table 4:** APE values were compared between our proposed method and RTABMAP as well as ORB-SLAM2 in Dataset V1_03_difficult.

Dataset ID	Method	Std	RMSE	Min	Median	Mean	Max
V103	RTABMAP-VIWO	0.06715	0.11993	0.02001	0.08238	0.09937	0.56543
	RTABMAP	0.16965	0.27017	0.01908	0.14518	0.21027	0.73483
	ORB-SAM2	0.10144	0.15709	0.01212	0.08991	0.11994	0.49687

**Table 5:** APE values were compared between our proposed method and RTABMAP as well as ORB-SLAM2 in Real Environment.

Test ID	Method	Std	RMSE	Min	Median	Mean	Max
Test 1	RTABMAP-VIWO	0.03236	0.05736	0.00430	0.03713	0.04736	0.21107
	RTABMAP	0.03411	0.11640	0.02933	0.11352	0.11129	0.19791
	ORB-SAM2	0.16599	0.39933	0.06341	0.36843	0.36320	0.98348
Test 2	RTABMAP-VIWO	0.03336	0.06411	0.00685	0.04551	0.05475	0.14472
	RTABMAP	0.09341	0.22949	0.02395	0.19688	0.20961	0.39448
	ORB-SAM2	0.17345	0.34326	0.01885	0.29120	0.29621	0.84962
Test 3	RTABMAP-VIWO	0.22203	0.36744	0.04565	0.22955	0.29277	0.78441
	RTABMAP	0.37641	0.51839	0.01877	0.18227	0.35644	1.38872
	ORB-SAM2	0.88130	1.50840	0.25462	0.88784	1.22417	4.34613

## Data Availability

The EuRoC MAV Dataset is available online at https://projects.asl.ethz.ch/datasets/doku.php?id=kmavvisualinertialdatasets (accessed on 3 August 2023).

## References

[1] Belter D., Nowicki M.R. (2019). Optimization-based legged odometry and sensor fusion for legged robot continuous localization. Robot. Auton. Syst..

[2] Garcia-Fidalgo E., Ortiz A. (2015). Vision-based topological mapping and localization methods: A survey. Robot. Auton. Syst..

[3] Angladon V., Gasparini S., Charvillat V. (2019). An evaluation of real-time RGB-D visual odometry algorithms on mobile devices. J. Real-Time Image Process..

[4] Han Y., Tang C., Xiao K. (2023). RGB-D Dense Map Construction Based on Improved ORB-SLAM2 Algorithm. J. Hunan Univ..

[5] Guclu O., Can A.B. (2019). Fast and effective loop closure detection to improve SLAM performance. J. Intell. Robot. Syst..

[6] Wang Z., Wu Y., Niu Q. (2020). Multi-Sensor Fusion in Automated Driving: A Survey. IEEE Access.

[7] Mourikis A.I., Roumeliotis S.I. A Multi-State Constraint Kalman Filter for Vision-Aided Inertial Navigation. Proceedings of the 2007 IEEE International Conference on Robotics and Automation.

[8] Li Y., Tang X., Li Z. (2021). Multi-sensor information fusion for mobile robots. J. Northwestern Polytech. Univ..

[9] Shi J., Zha F., Sun L. (2020). A Survey of Visual-Inertial Slam for Mobile Robots. Robot.

[10] Bloesch M., Omari S., Hutter M., Siegwart R. Robust visual inertial odometry using a direct EKF-based approach. Proceedings of the 2015 IEEE/RSJ International Conference on Intelligent Robots and Systems (IROS).

[11] Bloesch M., Burri M., Omari S., Hutter M., Siegwart R. (2017). Iterated Extended Kalman Filter Based Visual-Inertial Odometry Using Direct Photometric Feedback. Int. J. Robot. Res..

[12] Leutenegger S., Lynen S., Bosse M., Siegwart R., Furgale P. (2014). Keyframe-Based Visual–Inertial Odometry Using Nonlinear Optimization. Int. J. Robot. Res..

[13] Qin T., Li P., Shen S. (2018). Vins-mono: A robust and versatile monocular visual-inertial state estimator. IEEE Trans. Robot..

[14] Mur-Artal R., Montiel J.M.M., Tardós J.D. (2015). Orb-Slam: A Versatile and Accurate Monocular Slam System. IEEE Trans. Robot..

[15] Mur-Artal R., Tardós J.D. (2017). Orb-slam2: An open-source slam system for monocular, stereo, and RGB-D cameras. IEEE Trans. Robot..

[16] Stumberg L.V., Cremers D. (2022). DM-VIO: Delayed Marginalization Visual-Inertial Odometry. IEEE Robot. Autom. Lett..

[17] Liu M., Tao Y., Wang Z. (2023). Research on Simultaneous localization and mapping Algorithm based on lidar and IMU. Math. Biosci. Eng..

[18] Labbé M., Michaud F. (2019). RTAB-Map as an open-source lidar and visual simultaneous localization and mapping library for large-scale and long-term online operation. J. Field Robot..

[19] Ban C., Ren G., Wang B. (2020). Research on self-adaptive EKF algorithm for robot attitude measurement based on IMU. Chin. J. Sci. Instrum..

[20] Wang J., Xu S., Cheng N., You Y., Zhang X., Tang Z., Yang X. (2015). Orientation Estimation Algorithm for Motion Based on Multi-Sensor. Comput. Syst. Appl..

[21] Duan X., Jiang W., Yang G. (2018). Research on calibration testing method of ADIS16488 MEMS IMU. J. Test Meas. Technol..

[22] Teng Z., Han B., Cao J. (2023). PLI-SLAM: A Tightly-Coupled Stereo Visual-Inertial SLAM System with Point and Line Features. Remote Sens..

[23] Jiang C., Chen S., Chen Y. (2018). A MEMS IMU de-noising method using long short term memory recurrent neural networks (LSTM-RNN). Sensors.

[24] Gao Y., Shi D., Li R. (2022). Gyro-Net: IMU Gyroscopes Random Errors Compensation Method Based on Deep Learning. IEEE Robot. Autom. Lett..

[25] Xu Q., Gao Z., Yang C. (2023). High-Accuracy Positioning in GNSS-Blocked Areas by Using the MSCKF-Based SF-RTK/IMU/Camera Tight Integration. Remote Sens..

[26] Jiang X., Zhang X., Li M. (2017). Random Error Analysis Method for MEMS Gyroscope Based on Allan Variance. J. Test Meas. Technol..

[27] Song H., Yang P., Xu L. (2013). Analysis and Processing on Stochastic Error of MEMS Sensor. Chin. J. Sens. Actuators.

[28] The Kalibr Visual-Inertial Calibration Toolbox. https://github.com/ethz-asl/kalibr.

[29] Imu_Utils: A Ros Package Tool to Analyze the IMU Performance. https://github.com/gaowenliang/imu_utils.

[30] Colonnier F., Della Vedova L., Orchard G. (2021). ESPEE: Event-Based Sensor Pose Estimation Using an Extended Kalman Filter. Sensors.

[31] Mallios A., Ridao P., Ribas D., Maurelli F., Pétillot Y.R. EKF-SLAM for AUV navigation under probabilistic sonar scan-matching. Proceedings of the 2010 IEEE/RSJ International Conference on Intelligent Robots and Systems.

[32] Yan Y., Zhang B., Zhou J., Zhang Y., Liu X. (2022). Real-Time Localization and Mapping Utilizing Multi-Sensor Fusion and Visual–IMU–Wheel Odometry for Agricultural Robots in Unstructured, Dynamic and GPS-Denied Greenhouse Environments. Agronomy.

[33] Robot_Localization: A Package of Nonlinear State Estimation Nodes. https://github.com/cra-ros-pkg/robot_localization.

[34] Huai Z., Huang G. (2022). Robocentric visual–inertial odometry. Int. J. Robot. Res..

[35] Jung J.H., Cha J., Chung J.Y. (2020). Monocular visual-inertial-wheel odometry using low-grade IMU in urban areas. IEEE Trans. Intell. Transp. Syst..

[36] Zhan H., Weerasekera C.S., Bian J.W., Reid I. Visual odometry revisited: What should be learnt?. Proceedings of the 2020 IEEE International Conference on Robotics and Automation (ICRA).

[37] EVO: Python Package for the Evaluation of Odometry and SLAM. https://github.com/MichaelGrupp/evo.

[38] Burri M., Nikolic J., Gohl P. (2016). The EuRoC micro aerial vehicle datasets. Int. J. Robot. Res..

